# An artificial intelligence-powered digital pathology platform to support large-scale deworming programs against soil-transmitted helminthiasis and intestinal schistosomiasis in resource-limited settings

**DOI:** 10.1371/journal.pntd.0013432

**Published:** 2026-03-18

**Authors:** Peter K. Ward, Mohammed Aliy Mohammed, Mio Ayana Heda, Lindsay A. Broadfield, Peter Dahlberg, Daniel Dana, Gemechu Tadesse Leta, Zeleke Mekonnen, Betty Nabatte, Narcis Kabatereine, Kristina M. Orrling, Sofie Van Hoecke, Bruno Levecke, Lieven J. Stuyver

**Affiliations:** 1 Department of Translational Physiology, Infectiology and Public Health, Ghent University, Merelbeke, Belgium; 2 IDLab, Ghent University - Imec, Zwijnaarde, Belgium; 3 Enaiblers AB, Uppsala, Sweden; 4 Institute of Health, Jimma University, Jimma, Ethiopia; 5 Ethiopian Public Health Institute, Addis Ababa, Ethiopia; 6 Division of Vector Borne and Neglected Tropical Diseases and Ministry of Health, Kampala, Uganda; 7 Lygature, Utrecht, The Netherlands; 8 Steering Committee Leader for AI4NTD Consortium, Beerse, Belgium; McGill University, CANADA

## Abstract

**Background:**

The World Health Organization (WHO) has emphasised the need for innovative diagnostic tools to support the control and elimination of neglected tropical diseases (NTDs). Microscopy-based diagnostics, the current standard, rely on trained technicians for labour-intensive processes, posing logistical challenges in the low-resource settings where NTDs are most prevalent. This study describes the technical details of an artificial intelligence-powered digital pathology (AI-DP) platform designed to support large-scale deworming programs for two NTDs, alongside its analytical performance and user experience in laboratory and field settings.

**Methodology/principal findings:**

The AI-DP platform integrates electronic data capture tools, whole-slide imaging scanners, onboard AI analysis, and result verification software to automate microscopy-based screening. Targeting soil-transmitted helminthiasis (STH) and intestinal schistosomiasis (SCH) as initial use cases, the system was deployed in Ethiopia and Uganda, scanning 951 Kato-Katz (KK) thick smears containing 43,919 verified helminth eggs. Using 5-fold cross-validation, precision/recall/average precision were 95.4%/91.7%/97.1% for *Ascaris lumbricoides*, 95.9%/86.7%/94.8% for *Trichuris trichiura*, 84.6%/86.6%/91.4% for hookworm, and 89.1%/79.1%/89.2% for *Schistosoma mansoni*. Feedback from 14 field users across 30 real-world scenarios indicated the AI-DP platform’s improved usability, particularly in hardware portability and software interfaces, though the average scan time of 12.5 minutes per smear was identified as a limitation.

**Conclusions/significance:**

The AI-DP platform demonstrates potential as a tool for efficient monitoring and evaluation of STH and SCH control programs by providing near-real-time data with quality controls. However, further validation studies are needed to assess its clinical diagnostic performance, field usability, and cost-effectiveness in large-scale STH and SCH deworming programs. Given that the platform also provides a pipeline for any microscopy-based diagnosis, its potential for other NTDs also needs further attention.

## Author summary

Intestine- and blood-dwelling parasitic afflict over a billion people in low-resource countries, yet diagnosis often depends on labour-intensive manual microscopy performed by trained technicians. We developed a portable, artificial intelligence–powered digital pathology (AI-DP) platform to automate this process, incorporating a field-deployable slide scanner, onboard AI for worm egg detection and identification in stool smears, and a user-friendly verification interface for technicians to review the results of AI. Designed with consideration of World Health Organization diagnostic needs for these diseases, the platform was tested in laboratory and zero-infrastructure field trials in Ethiopia and Uganda, and processed nearly 1,000 stool smears, detecting eggs of both intestinal-helminths and *Schistosoma mansoni* with over 90% precision and recall. Field users noted improvements in portability and ease of use, though scan times remain slower than manual microscopy. While the AI-DP platform shows potential as a tool for efficient monitoring and evaluation of worm control programs, with possible extension to other diseases, further validation studies are essential to evaluate its clinical diagnostic performance and cost-effectiveness in large-scale deworming initiatives.

## 1 Introduction

Neglected tropical diseases (NTDs) are a diverse group of parasitic, bacterial, viral and toxin-mediated conditions that collectively affect over 1.5 billion people [1]. These diseases disproportionately impact vulnerable populations in (sub)tropical countries with limited access to medical care and undermine both individual and community development. The World Health Organization (WHO) has identified the lack of accurate, reliable, affordable diagnostics as a major barrier to achieving the 2030 targets for NTD control and elimination [1]. To guide the development of new tools, WHO has published 18 target product profiles (TPPs) covering 13 NTDs, each outlining desired performance characteristics for future diagnostics [2].

Microscopy remains the most widely used method for diagnosing NTDs [1], including both soil-transmitted helminthiasis (STH; infections caused by *Ascaris lumbricoides*, *Trichuris trichiura*, and hookworms (*Necator americanus* and *Ancylostoma* spp.)) and schistosomiasis (SCH, infections by *Schistosoma mansoni* (intestinal SCH) and *S. haematobium* (urinary SCH)), which affect an estimated 1.5 billion and 251 million people, respectively [3,4]. For STH and intestinal SCH, the WHO-recommended Kato-Katz (KK) method is widely used to prepare stool smears for visual egg counting, providing data on both prevalence and infection intensity [5]. Although the KK method is inexpensive, simple, and rapid, it suffers from low sensitivity at light infection intensities, rapid degeneration of hookworm eggs (within one hour), and the requirement for trained technicians [1,6–8].

Beyond these technical limitations, KK-based surveys incur substantial personnel costs, accounting for 42–74% of total study expenses [9,10]. Moreover, personnel requirements scale with sample throughput and the use of duplicate or triplicate smears per participant, making labour the single largest cost component in STH and SCH monitoring programs [9–12]. As programs shift from morbidity control toward elimination as a public health problem, the need for higher-coverage surveys, rising salaries, and dwindling skilled capacity will further escalate operational costs [11–13]. In recognition of these challenges, WHO has emphasised the urgent need for improved diagnostics and workflows in its STH and SCH TPPs [14,15].

Given the limited prospects for non-stool-based methods by 2030 [13], automating aspects of microscopy to boost throughput and reduce technician burden represents a practical strategy. Recent efforts have focused on developing low-cost, automated optical devices integrated with artificial intelligence, hereafter referred to as artificial intelligence-powered digital pathology (AI-DP), to support monitoring and evaluation (M&E) of STH and SCH control programs [16–18]. These approaches leverage advanced imaging hardware and machine-learning algorithms with the aim to enhance diagnostic accuracy, scalability, and cost-effectiveness by reducing reliance on skilled personnel and accelerating data acquisition. However, these efforts still highlight the numerous technical, operational and regulatory hurdles to address WHO TPP criteria [16]. For example, our initial proof-of-concept AI-DP platform [19] suffered from low throughput, bulky mains-only hardware, inconsistent focus (exacerbated by variable smear thickness and slide quality), and labour-intensive, error-prone ground-truth annotation processes.

This study aims (i) to describe the technical details of the AI-DP platform that was designed based on the aforementioned lessons learned, to report on (ii) the analytical performance of the AI model when using the updated platform and (iii) the user experience when used in both a laboratory and field setting. Note that a formal protocol for clinical diagnostic performance evaluation and usability of this AI-DP platform has been published [20] and will be reported separately once results are available.

## 2 Methods

### 2.1 Ethics statement

The study was approved by the Jimma University Institutional Review Board (IHRPGD/555/21) in Ethiopia and by the Ugandan Ministry of Health Vector Control Division Research Ethics Committee (VCDREC154) in Uganda. Written informed consent was obtained from all adult participants and from parents or legal guardians of minors; children aged ≥12 years in Ethiopia and ≥8 years in Uganda provided written assent. Participant confidentiality was maintained via pseudonymised QR coded identifiers (see Data storage, access control, and transmission). Individuals diagnosed positive by manual KK were treated per national guidelines: single-dose albendazole (400 mg) for STH and praziquantel (40 mg/kg) for intestinal SCH.

### 2.2 AI-DP platform design

**Fig 1:**
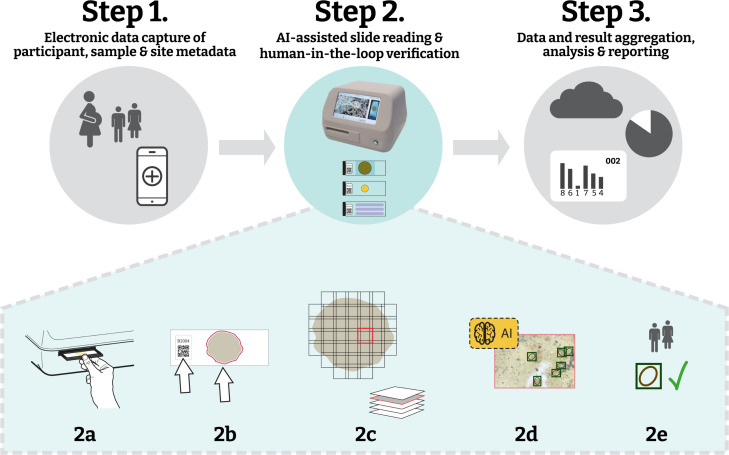
Overview of the AI-powered digital pathology (AI-DP) workflow. Step 1: electronic data capture of participant, sample and site metadata using a mobile app; Step 2: AI-assisted slide reading with human-in-the-loop verification, comprising (2a) loading a standard glass slide into the scanner, (2b) QR-code readout and automated detection of scan area, (2c) automated focus-stack acquisition across the specimen, (2d) onboard AI detection of helminth eggs, and (2e) human verification of AI detections; and Step 3: aggregation, analysis and reporting of results via a cloud-connected dashboard.

The platform was designed according to our AI-DP specific interpretation of the WHO TPPs for M&E of STH control programs [21]. In consultation with the AI4NTD consortium partners, we defined data workflows and product requirements to guide the platform’s design and development. Based on this consultation, the workflow of the AI-DP platform now consists of three major steps (Fig 1). In a **first step**, pseudonymised metadata of participants, samples and study sites were captured via an electronic data capture (EDC) tool available as an online/offline mobile application or as a web-based application, which handles participant registration, quick response (QR)-coded sample tracking, and offline caching, with secure synchronisation once connectivity is established.

In the **second step**, KK slides were automatically scanned, AI-detected helminth eggs underwent human-in-the-loop verification, after which per-species egg counts were generated. This step comprised the following operations: a barcode-labelled slide was loaded into the cradle of the whole-slide image (WSI) scanner (step **2a**), the QR code was read and the stool smear boundaries were automatically mapped (step **2b**), the WSI scanner captured focus-stacked image tiles across all fields of view (step **2c**). Each tile image was submitted to the AI model (a trained convolutional neural network) for egg detection and classification (step **2d**), and the resulting detections were presented in the EggInspector interface for technician confirmation or correction (step **2e**). This human-in-the-loop verification enhanced specificity of results before being exported or transmitted to the reporting dashboard for downstream analysis.

In the **third step**, a cloud-accessible reporting dashboard aggregated technician-verified egg counts alongside survey metadata, offered interactive visualisations of infection prevalence and intensity, and supported export of results locally or via an application programming interface (API) for direct integration with health information systems.

**Fig 2:**
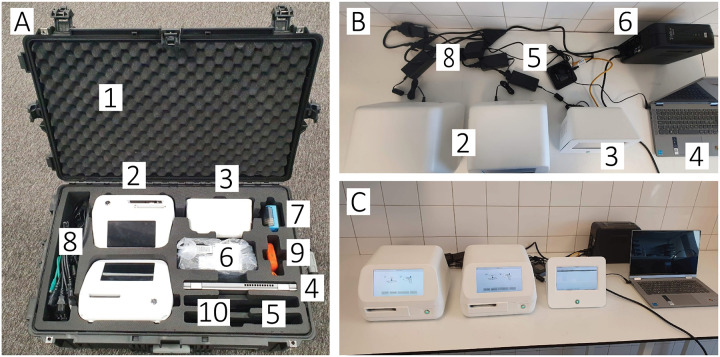
Field-deployable kit for the AI-powered digital-pathology (AI-DP) platform. Panel A: contents of the ruggedised transport case (total weight <32 kg), showing (1) a protective case with custom foam insert; (2) two whole-slide image (WSI) scanners; (3) slide-manager unit for local storage (up to 1,000 scans) and AI processing; (4) laptop computer; (5) Wi-Fi router; (6) uninterruptible power supply (UPS); (7) label printer; (8) network and power cables; (9) 4 TB USB external hard drive; (10) user manuals. Panel B: top view of unpacked equipment with all connections: the two WSI scanners (2), slide manager (3), laptop (4), Wi-Fi router (5), UPS (6) and power/ethernet cables (8). Panel C: fully assembled system on the laboratory bench, ready for slide digitisation, data capture and on-site AI inference.

To cover both extremes of M&E survey settings and align with the TPP requirement for operation in lowest level infrastructure, we designed the AI-DP platform for school-based surveys in ‘zero-infrastructure’ locations having no grid power, running water, or internet, and laboratory-based surveys with intermittent access to utilities. We packed all necessary hardware into a single ruggedised case (gross weight <32 kg; see Fig 2), including (i) two portable WSI scanners, (ii) a Slide Manager server (local image repository and AI inference), (iii) a dedicated laptop for study management and human-in-the-loop verification of AI detections, (iv) uninterruptible power supply (UPS) with sealed lead-acid battery, (v) label printer, (vi) slide holders, (vii) power and data cables, (viii) Wi-Fi router for high-bandwidth local networking and (ix) printed user manuals. The case with foam inlay prevented the equipment from vibration-induced misalignment and damage during air travel or rough-road transport. In the following sections we provide more technical details on the most important components of the AI-DP platform.

#### 2.2.1 Electronic data capture.

**Fig 3:**
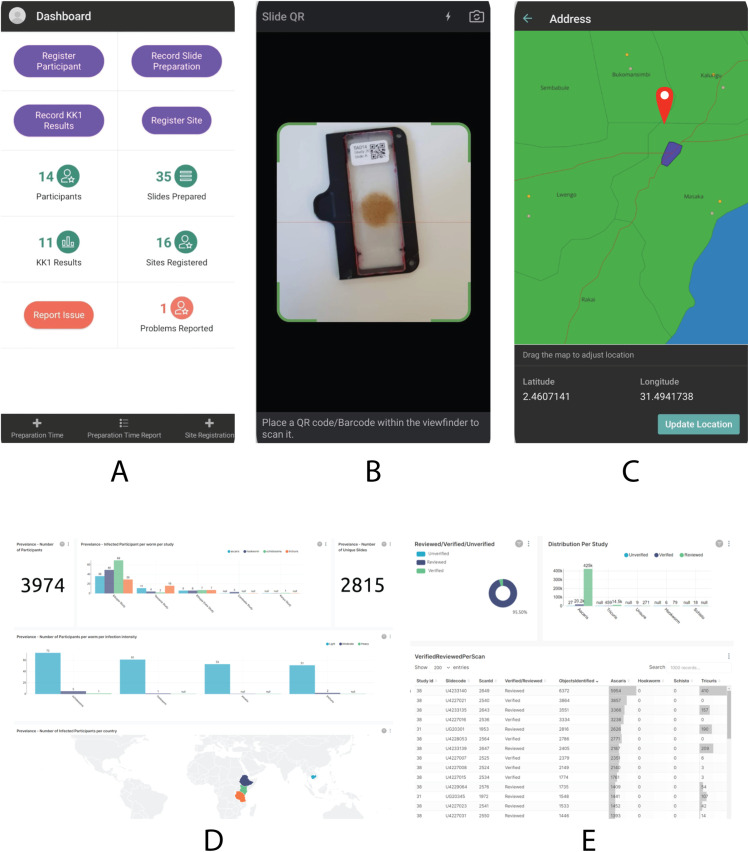
Electronic data capture (EDC) application and reporting dashboard. Panel A: customisable main screen of the mobile EDC app for registering participants and study sites, and collecting metadata such as human-microscopy Kato-Katz egg counts. Panel B: built-in QR-code scanner to timestamp events such as slide-preparation. Panel C: geolocation interface for mapping sample-collection sites, with adjustable pin and latitude/longitude display (map base layer: Natural Earth 1:10m Admin 0 – Countries; public domain). Panel D: web-based dashboard presenting real-time data summaries, prevalence charts and overall study-progress metrics, implemented using Apache Superset (Apache‑2.0 license) and displaying geographic distributions using Natural Earth country boundaries (public domain). Panel E: detailed verification panel showing per-scan status (verified, reviewed, unverified) alongside egg-count results in tabular form, implemented using Apache Superset (Apache‑2.0 license).

To enable a fully digital, end-to-end workflow, the AI-DP platform assigned a unique QR code label to every sample container, slide, and result form. A mobile EDC app scanned these labels (Fig 3) and automatically linked each sample identifier to timestamps (collection, preparation, and slide-reading), study site registration details and participant demographics, geospatial coordinates, and optionally water, sanitation, and hygiene (WASH) indicators. Only pseudonymised identifiers (e.g., study‑specific participant IDs) were stored digitally; no direct personal identifiers were captured within the EDC application or scanner software. Although the EDC application supports encryption of fields containing personally identifiable information at rest, this functionality was not used in the present study because no such identifiers were collected digitally.

Data could be captured offline and later synchronised to a central or cloud‑based repository, and exchanged with established EDC systems, such as Open Data Kit (ODK) and District Health Information System 2 (DHIS2), via application programming interfaces (APIs), ensuring interoperability with national health information systems. The linkage key mapping participant IDs to personal information was maintained by local study managers on paper in a secure location in-country.

#### 2.2.2 Data storage, access control, and transmission.

During field operation, all data generated by the AI‑DP platform, including whole‑slide images, AI‑generated detections, verification results, and associated metadata, were stored locally on the WSI scanners and/or the Slide Manager. The platform was designed to operate fully offline, and no data transfer is required for image acquisition, AI inference, or result verification.

Access to locally stored data was restricted through operating‑system and application‑level controls, including authenticated access to databases and verification tools. Personally identifiable information was not stored digitally within the platform; linkage keys mapping study‑specific identifiers to participant identities were maintained separately by local study teams in accordance with ethical approvals.

When connectivity was available, selected data and results could be transmitted between scanners, mobile devices, and cloud‑based dashboards using encrypted communication protocols. Upload of image data was optional and user‑controlled. In low‑connectivity settings, data can alternatively be exported to external storage for later synchronisation.

#### 2.2.3 Whole-slide image scanner.

We designed the custom WSI scanner for two-button operation (‘Load slide’ and ‘Start scan’). Standard 75 x 25 mm glass slides were clamped into a magnetic holder on a motorised XY stage. A secondary overview camera read the slide’s QR code, confirmed the sample type and outline of the KK thick smear, and presented the scan region to the user for confirmation. Operators could adjust the scanning region manually if required and then proceed to start the scan. During scanning, the stage advanced the slide through the region while a high-resolution camera captured images at every field of view within the determined boundary.

Imaging was performed with a 1/2.6-inch, 2-megapixel CMOS sensor (3x3 μm pixels) coupled to an inverted lens (7.5 mm focal length, 32.5 mm back focal length) yielding 3.2x magnification, a 1,280 x 720 μm field of view, a single pixel resolution of 0.93 μm, and a theoretical optical resolution of 1.2 μm. This provided an optical resolution comparable to traditional brightfield microscopy with a 10x/0.25NA Plan Achromat objective. The camera streams at 120 frames per second with illumination being provided by an LED source and diffuser. Captured images could be digitally enlarged via the verification tool.

KK thick smears typically spanned 20 mm in diameter and 135 μm in thickness (from 41.7 mg stool) with local thickness variations. To ensure at least one in-focus image per field of view, the scanner captured Z-stacks of 8 images at 20 μm intervals (total depth 240 μm) using a liquid lens (having ±0.67 mm range and 2.8 μm step size). A Sobel-filter focus metric ranked each stack; the single sharpest image was processed by the AI model for helminth egg detection and classification. Users could also perform manual refocusing post-scan, analogous to standard microscope workflows.

The WSI scanners were powered by direct current (DC) 19 V from common laptop chargers for continuous operation, or off a 19V 27,000 mAh USB power bank for up to 3 hours. The WSI scanners included a 1 TB drive permitting approximately 1,000 scanned slides. On-board AI inference ran on an NVIDIA Jetson Xavier NX in a standalone setup or on an NVIDIA Jetson Nano in a high throughput scenario when paired with a Slide Manager (discussed further under AI development).

#### 2.2.4 AI result generation.

Image processing ran either on-board each WSI scanner in standalone mode or centrally on a Slide Manager when multiple scanners were deployed. The Slide Manager, powered by an NVIDIA Jetson AGX Xavier, provided substantially more compute than the Jetson Nano modules in individual scanners. In centralised operation, images from up to five scanners could stream wirelessly to the Slide Manager, which performed AI inference, consolidated storage, and delivered near-real-time results. This architecture simplified data management, quality control, and remote verification. During development tests, five scanners ran in parallel under one Slide Manager, while in field deployments in Uganda and Ethiopia, we routinely paired two scanners with a single Slide Manager.

During scanning, each field of view image was sent to the Slide Manager, where an offline AI model automatically detected, classified, and quantified helminth eggs as images were acquired. We followed the KK thick smear protocol to convert raw egg counts to eggs per gram (EPG) [5]. Field of view images included a 10% overlap (≈100 µm) in both axes to ensure full smear coverage and reduce edge artifacts. To prevent double-counting, we grouped duplicate detections across overlapping fields using k-means clustering based on spatial proximity and type similarity. We then categorised EPG values as negative, light, moderate, or heavy infection according to WHO thresholds [23]. Per species results were available immediately in the local interface and, when internet connectivity is available, can be optionally uploaded, with or without accompanying images to a cloud-based dashboard for further analysis and remote verification.

#### 2.2.5 Result verification and data annotation tool.

**Fig 4:**
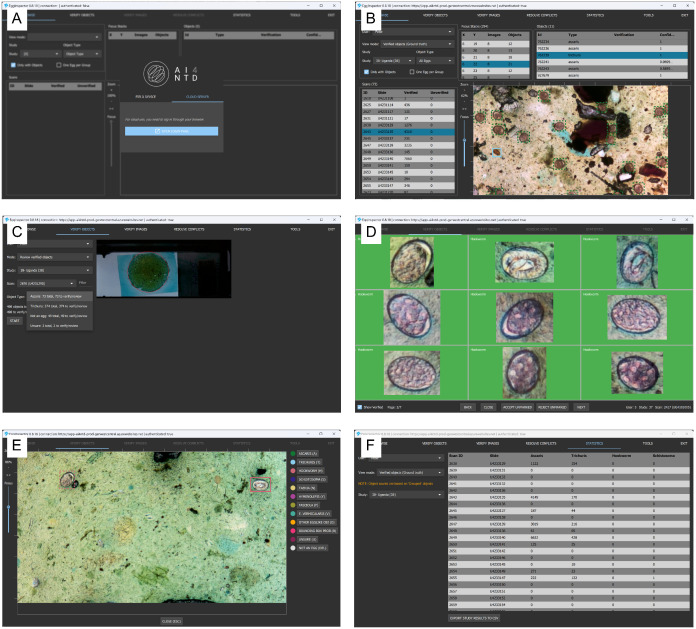
EggInspector, a human-in-the-loop AI verification and image annotation tool. **Panel A: ‘**Browse’ page lists scanned slides with selectable fields of view, detection-status filters (by species or verification status) and table of candidate detections. **Panel B:** ‘Verify Objects’ page displaying a selected scan with options to filter objects for verification, and a summary of the verification status. **Panel C:** batch verification grid (3x3 cropped thumbnails) of unverified *Ascaris lumbricoides* eggs, with Accept/Reject/Reclassify controls. **Panel D:** batch verification grid of verified hookworm eggs. **Panel E:** Contextual view of a selected hookworm egg within the full field of view, with all other detected helminth eggs highlighted and controls for z-stack focus adjustment and image adjustments. **Panel F:** summary page reporting total egg counts by species, eggs per gram (EPG) and options to export results locally or via the central dashboard.

To support diagnostic validation and continuous AI improvement, we developed EggInspector, an application that allowed for human-in-the-loop verification and object annotation. The application was available in both offline (local field) and online (cloud-synchronised) modes. On launch (Fig 4A), users choose between local data or previously synchronised studies. The Browse tab (Fig 4B) lists scanned slides, their fields of view, and detection filters (by helminth species or verification status). In the ‘Verify Objects’ view (Fig 4C), a slide overview showed AI detection overlays alongside a species-specific list of candidate helminth eggs. Verification proceeded in a 3x3 grid of cropped thumbnails (Fig 4D), one species at a time; users may accept, reject, or reclassify each detection. Selecting any thumbnail opened the full field of view image (Fig 4E), highlights all detections, and enabled focal-plane adjustment via the captured z-stack.

Once verification is complete, EggInspector allowed users to aggregate species-level egg counts and export summaries to local storage or via cloud upload (Fig 4F). This hybrid AI-technician, human-in-the-loop workflow is expected to enhance diagnostic specificity by enabling users to visually verify AI-detected positives and eliminate false positives, thereby increasing confidence when ‘ruling in’ the presence of eggs. Optional manual annotation of missed eggs during model-training sessions can further increase diagnostic sensitivity by reducing false negatives.

#### 2.2.6 Data verification and reporting.

After technician verification via EggInspector, per-species egg counts were generated and converted to EPG using the standard KK thick smear protocol [5]. Light, moderate, or heavy infection categories per species were assigned using WHO thresholds [22]. Data exports included verified egg counts by species, slide-level EPG values, intensity thresholds, associated images, and metadata. Exports could be stored locally, or when internet access was available, automatically synchronised to a cloud-based dashboard. This setup enabled near-real-time visualisation of study progress, and facilitated secondary remote verification by expert reviewers. In low-connectivity settings, completed scan datasets were exported to external storage devices (e.g., USB drives) and uploaded to central servers once a connection was established.

### 2.3 Analytical performance of artificial intelligence

The analytical performance of our AI model was assessed through four stages: scanning of KK thick smears, ground truth dataset creation, model training and selection, and statistical data analysis.

#### 2.3.1 Scanning of KK thick smears.

Stool samples were collected in two NTD endemic countries: Ethiopia (Jimma town) and Uganda (West Central and West Nile regions). In Ethiopia, we recruited students aged 5–15 years (balanced by sex) from eleven primary schools. In Uganda, we enrolled schoolchildren (5–14 years) and community members aged ≥6 months. Each participant provided one stool sample in a labelled container. Samples from Jimma were transported at ambient temperature to the university laboratory; in Uganda, preparation of the KK thick smears, microscopy, and slide scanning were performed onsite under zero-infrastructure conditions (typically no running water, electricity, or internet).

Stool specimens were processed using the WHO KK method [5]. In brief, approximately 41.7 mg of sieved stool was transferred through a standard template onto a glass slide, covered with a glycerol–malachite green–soaked cellophane strip, and pressed flat. Each slide received a preprinted QR label encoding slide ID (unique pseudonymised identifier mapped to participants in a given study, a key of the mappings was stored on paper and managed by the local study coordinators), sample type ID (KK), study ID, date, and time stamp for automated tracking by the WSI scanner.

To build the ground‑truth dataset for AI training, we purposely scanned only KK slides confirmed positive for STH or intestinal SCH eggs by conventional microscopy. This approach minimised interruption of ongoing surveys and reduced data annotation effort while still providing large egg-free areas and common artefacts within each slide. Negative-control slides for estimating slide‑level specificity in diagnostic comparisons will be included in later validation studies described in [20]. Slides were typically scanned 30–60 minutes after preparation to capture hookworm eggs before degeneration. As manual microscopy within the survey took priority, some slides were scanned up to 24 hours later for improved *S. mansoni* egg visibility [5].

#### 2.3.2 Ground truth dataset creation.

We assembled a high-quality WSI dataset with image tiles organised by slide ID. Each image record included slide and image IDs, species class labels and bounding box coordinates (x_min, y_min, x_max, y_max). Annotation proceeded in three phases to ensure label accuracy: (i) eight parasitologists independently drew bounding boxes and assigned helminth species labels in EggInspector; (ii) the same parasitologists then provided a second review for images they had not previously seen during their first round of annotation. During this review, verifiers could do one of the following:

- accept the image, confirming all visible labels and acknowledging that no visible eggs were missing,
- change visible objects to an ‘Unsure’ label if uncertain, reject individual objects if confident they were not helminth eggs,
- reassign a label to another helminth type (e.g., *Hymenolepis*, *Taenia*, *Fasciola*, or an ‘Other’ category for unspecified helminths), while identifying and annotating any omissions.

In case of disagreements, (iii) a third parasitologist adjudicated any disagreements to reach a consensus by reviewing only the objects in conflict. Here too, the adjudicator could accept, reject, mark the object as unsure, or change the label type. Images with unresolved conflicts, meaning no majority consensus was achieved on the object label even after review by multiple verifiers (e.g., differing opinions on species classification or whether an object is an egg), were excluded from the ground truth dataset until a conflict resolution process, such as a majority vote on the label, could be completed. Only images verified by at least two parasitologists with agreement on the labelled objects were retained for training, ensuring reliability and consistency in the dataset.

#### 2.3.3 Model training and selection.

To prevent data leakage, we employed a grouped five-fold cross-validation strategy (implemented with scikit-learn’s GroupKFold [23]), ensuring all images from the same slide (and participant) were assigned to a single fold. Each fold was trained on four partitions and evaluated on the held-out partition. We applied transfer learning with the pretrained YOLOv8n model (COCO weights) [24], selecting the nano variant for its compact size and fast inference capabilities on embedded hardware. Training was initialised for 100 epochs per fold on a Tesla T4 graphics processing unit (GPU, 15 GiB) using Python 3.10.13 and PyTorch 2.1.2, using the default Ultralytics YOLOv8 hyperparameters with early stopping to prevent overfitting. The epoch with the highest Ultralytics evaluation score was selected as the best model for each fold. The code and referenced image dataset used in this study are available via Kaggle [25,26].

#### 2.3.4 Statistical analysis.

For each fold and each helminth species, we computed the following metrics at the object level:

Precision (positive predictive value), defined as


$$Precision=\frac{True\;Positives}{True\;Positives+False\;Postives};\mathrm{\backslash ]}$$

Recall (sensitivity or true positive rate), defined as


$$Recall=\frac{True\;Positives}{True\;Positives+False\;Negatives};\mathrm{\backslash ]}$$

Average precision at an intersection over union of 0.50 (AP50), defined as the area under the precision–recall curve at a 50% overlap threshold [27].

Due to the design of our dataset, which exclusively targeted smears confirmed positive for STH or SCH to maximise annotation of helminth eggs for initial model training, we lacked negative-control slides (i.e., slides confirmed free of helminth eggs) necessary to compute diagnostic specificity or negative predictive value. While object-level precision, recall and AP50 characterise the model’s per-egg detection performance, slide-level specificity, repeatability and reproducibility will be assessed in future work incorporating egg-free specimens [20].

### 2.4 User experience

To evaluate the user experience of the platform, informal user feedback, operational observations and written feedback were gathered from users.

## 3 Results

### 3.1 AI performance

We scanned 951 KK thick smears in this study (Ethiopia: n = 331; Uganda: n = 620), producing 1,156 whole-slide images (Ethiopia: 520; Uganda: 636). Slides were included only if conventional microscopy confirmed the presence of eggs. From these scans, we extracted 8,695 field of view images that each contained at least one helminth egg. In total, 43,919 egg annotations were verified independently by two parasitologists. Species-level distribution was as follows: *A. lumbricoides* (38,854 eggs; 88.5%), *T. trichiura* (3,220 eggs; 7.3%), hookworm (548 eggs; 1.2%), and *S. mansoni* (1,297 eggs; 3.0%). This curated, expert-verified dataset formed the ground truth for model training and cross-validation.

We evaluated detection and classification performance using five-fold GroupKFold cross-validation with slide-based grouping, as detailed in the Methods section. Table 1 reports the mean ± standard deviation (SD) of precision, recall, and AP50 across the five folds; the full fold-wise breakdown is provided in S1 Table.

**Table 1:** Summary of AI model performance with k-fold cross-validation across 8,695 images.

			Average performance across folds		
		Total Eggs	Precision ± SD.	Recall ± SD	AP50 ± SD
**Cross-validated means**	***A. lumbricoides***	38,854 (88.5%)	95.4% ± 2.1%	91.7% ± 5.8%	97.1% ± 2.3%
	***T. trichiura***	3,220 (7.3%)	95.9% ± 0.9%	86.7% ± 3.3%	94.8% ± 1.2%
	**Hookworm**	548 (1.2%)	84.6% ± 8.0%	86.6% ± 8.5%	91.4% ± 5.4%
	***S. mansoni***	1,297 (3.0%)	89.1% ± 3.3%	79.1% ± 8.6%	89.2% ± 3.8%
**Mean across classes**		43,919	91.3% ± 5.4%	86.1% ± 5.2%	93.1% ± 3.5%

Mean ± standard deviation (SD) of precision, recall and average precision at an intersection over union of 0.50 (AP50) per helminth species over five-fold GroupKFold cross-validation. The mean performance across species is also shown.

While the precision (>84%), recall (>79%) and AP50 (>91%) were high for all helminth species, the model performed best for *A. lumbricoides* and *T. trichiura*. Hookworm had the lowest precision (84.6%), while *S. mansoni* showed the lowest recall (79.1%) and AP50 (89.2%). For field deployment, we retrained the YOLOv8n model on the full annotated dataset for 70 epochs (the average of the optimal epochs from each cross-validation fold). The frozen model was exported in ONNX format and distributed to edge devices via over-the-air updates. Benchmarking on 1,000 sample images yielded mean inference times of 240 ms/image on NVIDIA Jetson AGX Xavier, 234 ms/image on NVIDIA Jetson Xavier NX, 770 ms/image on NVIDIA Jetson Nano. These results demonstrated that the platform could perform near–real-time analysis even on resource-constrained hardware.

### 3.2 User experience

**Fig 5:**
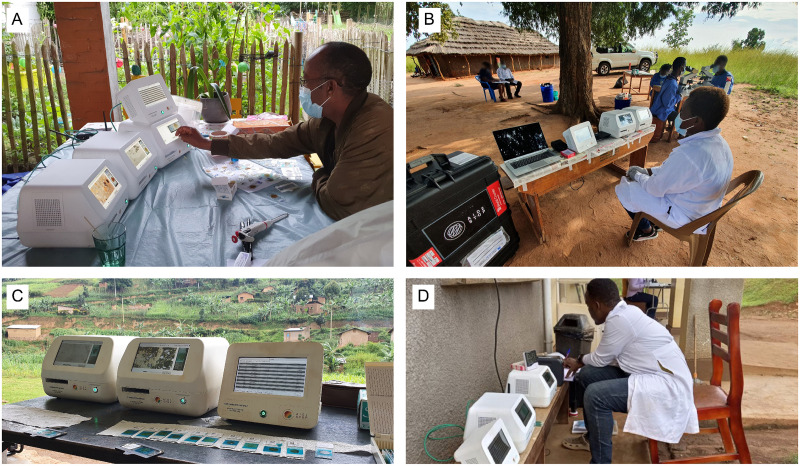
The artificial intelligence-powered digital pathology (AI-DP) platform was tested in various field settings and conditions. **Panel A:** simulated field testing and system training in Belgium, by members from Jimma University and Ghent University. Additional testing was performed in laboratory environments at both Jimma University and Ghent University. **Panels B-D:** the AI-DP platform was transported to and evaluated in over 30 zero-infrastructure field sites, across six districts throughout Uganda by the Vector Control Division, Ministry of Health (Uganda), to perform data collection, evaluate the system in remote field settings, test digital workflows, and test integrated AI and data verification workflows. At each site slides were read first by traditional microscopy to screen for positive samples, which were then read by the AI-DP platform to support rapid build-up of the database for AI training.

During the development of the platform, several versions of the system were evaluated by 14 users across the two countries and a range of diverse settings. These settings included standard laboratory environments, simulated field conditions (Fig 5A), and over 30 real-world field scenarios (Fig 5B-5D).

The user observations/feedback and design recommendations for the AI-DP platform are summarised in Table 2. We grouped the observations feedback and the corresponding recommendations into five themes, including (i) hardware usability, (ii) power and portability, (iii) scanning performance, (iv) software and interface and (v) training and support. Generally, the AI-DP platform demonstrated a high degree of adaptability operating effectively in both laboratory environments and remote, off-grid locations.

**Table 2:** Summary of User Feedback and Design Recommendations for the AI-DP Platform.

Theme	Observations/ Feedback	Recommendations
**Hardware usability**	• Rugged case was reliable but bulky and heavy • Users expressed concern about conspicuous appearance of equipment case, which may attract undue attention in transit	• Maintain protection, consider weight/volume reduction • Adapt the outward appearance of a standard suitcase to reduce perceived value
	• On first use, it is not clear that magnets secure the slide	• Redesign tray for more tolerances of standard slides
	• Scanners had no handles, made lifting out of case difficult	• Add a handle to improve portability
	• Cable clutter due to external router and loose connections	• Integrate router, battery backup, and Slide Manager into scanner housing
**Power & portability**	• UPS sustained short blackouts; voltage fluctuations reduced reliability in off-grid sites	• Include voltage stabilisers or more robust power regulation
	• Lithium power banks provided longer operation time, in a much smaller package and could be charged meanwhile	• Standardise around power banks and power input • Clarify air transport guidance • Enable hot swapping of power banks • Test with solar recharging
**Scanning performance**	• Initial scan time of 25–35 min/slide was too slow, but later improvement to around 10 min/slide makes it acceptable	• Optimise scanning algorithm and optics to reduce scan time further
	• One scanner could not be operated due to failed glue on magnetic pickup for slides	• Provide maintenance training, repair manuals, and local repair kits
**Software & interface**	• User interface effective for training; needs summary dashboards and high-contrast mode	• Add offline dashboards, help cues, and display options for field use
	• Manual annotation best with mouse in office setting	• Improve annotation tools for touchscreen/glove compatibility
**Training & support**	• Hands-on training better than paper manual	• Expand training programs and provide local biomedical support materials and videos
	• Remote support useful; field maintenance challenging	• Develop a printable/online troubleshooting manual • Review options for local support technicians

#### 3.2.1 Hardware usability.

Although users considered the equipment transport case bulky, it provided robust protection for the equipment during air and ground transport. Fieldwork in Uganda required long daily commutes over bumpy and challenging dirt roads to reach various schools, where the equipment was set up and studies were conducted. Users expressed concern about the conspicuous appearance of the case, noting it could attract undue attention during transit. Additionally, on first use, it was unclear to some users that magnets secured the slide in the scanner tray, leading to potential handling issues. The lack of handles on the scanners made lifting them out of the case difficult, hindering setup efficiency. Lastly, cable clutter from an external router and loose connections was reported as a usability challenge in field settings. Recommendations to address these issues include reducing the case’s weight and volume while maintaining protection, adapting its outward appearance to resemble a standard suitcase to lower perceived value, redesigning the tray for greater tolerance of standard slides, adding handles to improve scanner portability, and integrating the router, battery backup, and Slide Manager into the scanner housing to minimise cable clutter.

#### 3.2.2 Power and portability.

In laboratory settings, the UPS could sustain operations during power interruptions for up to 2.5 hours. For continuous use over an entire working day (8 hours) at field sites lacking mains power, portable diesel generators were predominately used. However, voltage fluctuations from these generators reduced the reliability and lifespan of the UPS in off-grid environments. Lithium power bank batteries were later found to be a more affordable alternative, offering longer operation times (up to 3.5 hours on a 20,000 mAh capacity) with significantly smaller volume and weight, and the ability to be charged simultaneously from common power sources including USB-C chargers or solar panels. Nevertheless, these batteries presented restrictions during air transport for field trials, as they were only permitted in carry-on luggage and not in checked baggage. Recommendations to address these challenges include incorporating voltage stabilisers or more robust power regulation to mitigate fluctuations, standardising around power banks for power input, clarifying air transport guidance, enabling hot swapping of power banks, and testing solar recharging options.

#### 3.2.3 Scanning performance.

Users found the equipment setup straightforward, enabling them to assemble and initiate slide scanning in under 30 minutes with minimal guidance, training, or instructions. However, initial scan times of 25–35 minutes per slide were deemed too slow for efficient fieldwork, although later improvements reduced this to an average scan time of 12.5 (±6 SD) minutes per slide, with variability due to sample preparation and sample area with 622 (±102 SD) fields of view images per scan. While feedback from both countries emphasised a desire for faster scan times, stakeholders also valued the potential to parallelise scanning and automate data reporting and quality checks, which would free operators to focus on other tasks and improve overall cost efficiency. Additionally, hardware issues were noted, including one scanner becoming inoperable due to failed glue on the magnetic pickup for slides, preventing scanning operation. Recommendations include optimising the scanning algorithm and optics to further reduce scan time, as well as providing maintenance training, repair manuals, and local repair kits to address hardware failures. Other challenges observed during fieldwork, though not directly in Table 2, included susceptibility to insects entering WSI scanners during nighttime operations due to attraction to the bright light source, highlighting the need for an automatic closing flap on the slide loading port, and failures of Ethernet cables due to repeated setup and pack-down in outdoor environments, which were later mitigated by a software update enabling wireless image transmission.

#### 3.2.4 Software and interface.

Users initially performed manual annotations of helminth eggs using the EggInspector application on a laptop, a laborious but essential task for developing the database for AI model training. This process was better suited to a laboratory or office environments and was most effective with a mouse due to the precision required to draw bounding boxes around objects; otherwise, users from both sites found the application user-friendly and easy to use. Once the AI model was trained, reviewing AI-detected objects on the laptop became more streamlined, focusing only on the egg-like objects. However, challenges included the lack of a high-contrast mode on the screen, which was problematic when operating in direct sunlight. Additionally, while the touch screens were capable of operation with disposable gloves, switching between tasks such as sample preparation, slide loading, and egg verification required careful consideration to glove disposal and equipment sterilisation. Users noted that verification work could be deferred to a later stage (e.g., after returning from the field) since images and data were preserved. Although laptop battery life was sufficient to sustain a full day of work, verifying eggs in field scenarios was considered potentially more suitable on a mobile device.

#### 3.2.5 Training and support.

Users emphasised the value of practical training over written instructions, finding hands-on training sessions far more effective than paper manuals for mastering the AI-DP platform’s operation. While remote support was useful for troubleshooting, field maintenance remained challenging due to limited on-site resources and technical expertise. Recommendations include expanding training programs with local biomedical support materials and instructional videos, as well as developing a printable or online troubleshooting manual to assist with common issues. Additionally, options for engaging local support technicians should be explored to enhance field support capabilities.

## 4 Discussion

In this study, we present (i) the technical details of the AI-DP platform designed based on lessons learned from field and laboratory evaluations, (ii) the analytical performance of the AI model, and (iii) the user experience in both controlled and real-world settings.

### 4.1 Advancing NTD monitoring: AI-DP enables near-real-time data with quality assurance and reduces technician burden

The AI-DP platform offers a promising approach to M&E of NTDs such as STH and intestinal SCH. By integrating WSI scanners, onboard AI analysis, and human-in-the-loop verification, it provides near-real-time, quality-assured data while reducing the burden on technicians. Iterative design improvements guided by earlier challenges [19], including reducing magnification from 10x to 3.2x for faster scanning, adopting high frame rate focus stack acquisition to handle uneven KK thick smear thicknesses, and optimising hardware for portability (e.g., rugged case under 32 kg, battery-powered operation). Compared to other devices in the field, such as those reviewed [16–18], our platform uniquely combines field-deployable hardware with offline AI inference and EDC, making it suitable for zero-infrastructure settings. Unlike many low-cost optical devices that prioritise cost and simplicity over throughput, our system aims to match or exceed manual microscopy turnaround times, though scan times (currently 12.5 minutes per slide) still lag behind expert manual reading (on average 6.8 minutes to read a single slide [10]). Nevertheless, automation frees technicians for parallel tasks, promising substantial labour savings – critical given that personnel costs constitute 42–74% of M&E survey expenses [9].

In addition to efficiency gains, the AI-DP platform offers operational advantages over human-only microscopy workflows. AI-DP detection is not affected by operator fatigue in the same way as human microscopists, whose accuracy and thoroughness can decline over long reading sessions. Provided that sample preparation quality is maintained throughout the day, the platform can deliver consistent performance over extended periods. Furthermore, its automated analysis and integrated verification pipeline reduce the risk of disruption from staff turnover or reassignment during extended surveys, a common challenge in large-scale monitoring programs. These factors can enhance reproducibility and reliability of results across time, sites, and operators.

### 4.2 Comparative AI approaches and analytical performance for STH/SCH

The precision (84.6–95.9%) and recall (79.1–91.7%) of our object detector are broadly consistent with published AI-DP approaches for the detection of STH and intestinal SCH [17,18]. Direct comparison across studies should be interpreted with caution, as they differ in datasets, annotation protocols, evaluation metrics (object- vs slide-level), optical configurations, and overall workflow*.*

Spotlab reported high cross-validation performance for *T. trichiura* (precision 98.44%, recall 80.94%) and combined *T. trichiura*/*A. lumbricoides* test-set performance (precision 94.36%, recall 93.08%), using a 3D-printed microscope and a mobile-phone microscope (Samsung S9, 2x, NA 0.13) with cloud inference. Their workflow included image upload (7 minutes at 2 Mbps for 7.2 x 7.2 mm^2^ scan area) and cloud processing (8 min) [28]. A pipeline using a Grundium Ocus 20 (6 MP, 20x objective, NA 0.4) with Aiforia’s cloud-based analysis reported sensitivities of 77–92% and specificities of 89–99% across similar parasites in field-collected Kato-Katz smears, with scanning time about 5–10 minutes and upload within 10–20 minutes at 5–8 Mbps; analysis of the WSIs with local hardware was estimated to take up to 2 hours [29]. In our earlier KK2.0 prototype (10x magnification) we observed a weighted mean precision of 94.9% and recall of 96.1% across four species [19]. The current platform uses lower magnification (3.2x), a larger dataset, and grouped five-fold cross-validation, yielding robust performance while markedly improving scanning speed.

Throughput and end-to-end time-to-result are critical for programmatic surveys. Based on reported scan, upload, and stitching times, a Spotlab-based workflow could require on the order of two hours per slide for a 20 mm Kato-Katz smear (e.g., ~ 30 minutes scanning, ~ 42 minutes upload, ~ 49 minutes stitching), and a Grundium plus Aiforia workflow ~20–35 minutes (scan 5–10 minutes, upload 10–20 minutes, analysis ~5 minutes at ~5–8 Mbps). By contrast, our platform performs on-device inference and avoids upload delays; with recent optimisations, typical scan times are 12.5 minutes per slide, enabling image analysis in parallel to scanning, and near-real-time review and verification in the field. A key operational distinction is that many AI-DP platforms rely on stable internet to upload images for cloud analysis and often lack integrated verification and end-to-end data capture [28,29]. Our platform combines automated sample detection, smear boundary mapping, fully offline AI inference, and human-in-the-loop verification with integrated EDC and reporting in a single workflow that requires minimal training. This integrated, offline-first design addresses technology-readiness and usability gaps identified in prior reviews [16,21] and advances alignment with WHO diagnostic TPPs for STH and SCH, especially for deployment in low- and zero-infrastructure [14].

### 4.3 Addressing ground truth data quality and class imbalance: key challenges in AI model performance

Despite the promising performance, challenges persist in our ground-truth database of 43,919 eggs from 951 slides. Imbalance in the helminth egg classes, particularly for underrepresented species such as hookworm (1.2% of eggs) and *S. mansoni* (3.0%), likely contributes to the lower recall observed for *S. mansoni* (79.1%) and the lower precision for hookworm (84.6%). Such class imbalance is a common limitation in AI-DP for helminth infections and reflects the epidemiological distribution encountered in endemic settings.

Equally critical is the quality of ground truth labels, as the annotation process requires rigorous and time-consuming verification effort by multiple parasitologists to ensure accuracy. Ideally, triple verification should be achieved for all images to maximise label quality. However, user fatigue during prolonged verification sessions poses an additional challenge, and may compromise the annotation consistency over time.

Future efforts will therefore prioritise expanded data collection for underrepresented species, alongside advanced AI techniques such as class weighting, custom data loaders, and data augmentation to mitigate class imbalance. Additionally, strategies to streamline verification, such as automated pre-annotation tools or user-friendly interfaces to reduce fatigue will be prioritised. Continued research into out-of-distribution performance will enhance model generalisability and robustness in diverse field conditions [30], ensuring consistent accuracy across varying sample qualities and preparation methods encountered during real-world deployment [27].

Importantly, our deployed approach intentionally combines object-level egg detection with human-in-the-loop confirmation, prioritising specificity, traceability, and auditability over fully autonomous diagnosis. This design choice reflects the intended use of the platform for programmatic monitoring and evaluation rather than individual clinical decision-making, and aligns with WHO target product profile requirements for quality assurance and data reliability in large-scale STH and schistosomiasis control programs.

### 4.4 Fostering country ownership: co-developing AI-DP through local user feedback for field settings

Collaboration with field users across Ethiopia and Uganda has been central to refining the AI-DP platform, emphasising country ownership and co-development to ensure relevance in resource-constrained settings. Feedback from 14 local users over 30 real-world field scenarios in these countries drove hardware improvements (e.g., integrating routers into scanner housing to reduce cable clutter, and adding handles for portability) and software enhancements (e.g., over-the-air updates to resolve Ethernet cable failures, and improved EggInspector interface with high-contrast mode for sunlight visibility). Engaging local technicians and researchers not only tailored the platform to address specific field challenges but also built capacity and fostered a sense of ownership among national stakeholders. Users noted that scan times remain a bottleneck compared to manual microscopy, prompting planned optimisations like AI-driven focus prediction to reduce Z-stack acquisition. Future developments, shaped by ongoing dialogue with local partners, include a mobile verification app for field convenience, streamlined QR printing workflows, and controlled manufacturing processes to ensure durability under harsh conditions. These iterative changes, grounded in a co-development approach, underscore the platform’s adaptability to the unique needs of endemic regions and its potential for sustainable integration into national health systems.

### 4.5 Validating performance: the critical next step for AI-DP adoption

While the current study focused on the technical design, the analytical performance and the user experience to inform iterative refinements, comprehensive validation studies are essential to establish its role in large-scale deworming programs. In Peru, a validation study demonstrated the AI-DP platform’s higher sensitivity when compared to manual microscopy for *A. lumbricoides* (77% *vs.* 57%), particularly at low infection intensities, and a comparable sensitivity for *T. trichiura* and hookworm [31]. These findings are in line with reports from other innovative optical devices for STH/SCH [16,31].

A detailed evaluation protocol [20] is underway in Ethiopia and Uganda, targeting school-age children to assess: (i) diagnostic performance, (ii) repeatability and reproducibility, (iii) time-to-result, (iv) cost-efficiency, and (v) usability in laboratory and field settings. This protocol aims to demonstrate non-inferiority against manual microscopy and aims to quantify efficiency gains and inform scalability. Integration with national health information systems raises regulatory, data-security, and privacy considerations that must be addressed for sustainable adoption. Sample preparation variability across sites further highlights the need for automated quality-control checks to reject substandard smears before scanning [20,31].

### 4.6 Exploring scalability: potential of AI-DP for broader NTD diagnostics

The modular design of the AI-DP platform and its adaptable AI-training framework position it as a versatile tool beyond STH and intestinal SCH. Low-hanging fruit includes adapting the system for urinary SCH (*S. haematobium*), where current magnification settings remain suitable, though urine filter samples may present challenges similar to those overcome with KK thick smears, such as variable thickness and the need for multiple focus planes to identify morphological features of eggs. Similarly, diagnostics for lymphatic filariasis using blood thick smears can leverage existing imaging parameters, as these smears are typically more uniform (monoplane) and microfilariae are larger than STH eggs, potentially simplifying detection. Other microscopy-based NTD diagnostics, such as mycetoma and scabies, could be incorporated with adjustments to AI models trained on relevant datasets and, if needed, minor tweaks to imaging protocols and magnification [32]. Beyond human health, the platform holds potential for One Health applications, supporting diagnostics in soil and animal health contexts in resource-limited settings, which could enhance the cost-benefit ratio of the scanner by broadening its utility across sectors. This scalability aligns with WHO’s 2030 NTD elimination goals [1], offering a unified diagnostic platform for resource-limited environments. Future work will explore these expansions, ensuring hardware and software flexibility to meet diverse diagnostic needs across human, animal, and environmental health domains.

## 5 Conclusions

The AI-DP platform offers a promising approach to field-deployable, AI-enhanced microscopy for STH and intestinal SCH M&E programs. By enabling near-real-time, quality-assured data and reducing the workload on technicians, it supports the potential for improved efficiency in STH and intestinal SCH control programs within endemic, resource-constrained settings. Its modular design and adaptable AI-training framework facilitate expansion to other microscopy-based NTD diagnostics, such as urinary schistosomiasis and lymphatic filariasis, as well as broader applications in One Health contexts, contributing to global elimination targets. However, comprehensive validation studies are essential to evaluate its clinical diagnostic performance, repeatability, cost-effectiveness, and usability across diverse field conditions in large-scale deworming programs, ensuring its readiness for sustainable implementation.

## Supporting information

S1 Table Summary of AI model performance with k-fold cross-validation. For each of the five GroupKFold cross-validation folds (images grouped by slide), precision, recall, and average precision at IoU = 0.50 (AP50) are reported ± standard deviation (SD) for each helminth species.
(PDF)
