# Supplementary material for: An artificial intelligence-powered digital pathology platform to support large-scale deworming programs against soil-transmitted helminthiasis and intestinal schistosomiasis in resource-limited settings
Source: PLoS Negl Trop Dis. 2026 Mar 18;20(3):e0013432. doi: 10.1371/journal.pntd.0013432 (PMC13016470; doi:10.1371/journal.pntd.0013432)
Supplement: S1 Table — For each of the five GroupKFold cross-validation folds (images grouped by slide), precision, recall, and average precision at IoU = 0.50 (AP50) are reported ± standard deviation (SD) for each helminth species. (PDF) [file pntd.0013432.s001.pdf]

S1 Table. Summary of AI model performance with k-fold cross-validation.

	Class	Images	Instances	Precision	Recall	AP50
**Fold 1**	***A. lumbricoides***	1,540	4,462	94.3%	94.0%	97.6%
	T. trichiura		748	97.5%	89.2%	96.1%
	**Hookworm**		143	91.1%	84.6%	92.9%
	S. mansoni		162	86.9%	81.9%	89.5%
**Fold 2**	***A. lumbricoides***	2,297	9,552	97.4%	94.4%	98.3%
	T. trichiura		882	95.6%	88.2%	95.0%
	**Hookworm**		157	77.5%	88.5%	91.3%
	S. mansoni		500	86.4%	71.4%	83.9%
**Fold 3**	***A. lumbricoides***	1,009	6,472	92.5%	81.8%	93.0%
	T. trichiura		515	96.0%	84.6%	95.6%
	**Hookworm**		58	75.1%	94.8%	95.0%
	S. mansoni		154	93.8%	69.0%	87.8%
**Fold 4**	***A. lumbricoides***	1,867	9,538	97.5%	91.9%	98.1%
	T. trichiura		611	95.6%	82.1%	92.9%
	**Hookworm**		78	86.6%	73.1%	82.2%
	S. mansoni		180	91.3%	88.9%	94.2%
**Fold 5**	***A. lumbricoides***	1,982	8,830	95.3%	96.4%	98.4%
	T. trichiura		464	95.0%	89.6%	94.6%
	**Hookworm**		112	92.8%	92.2%	95.5%
	S. mansoni		301	87.2%	84.4%	90.8%
